# Hemorrhage from Gun-Shot Wound

**Published:** 1861

**Authors:** 


### SELECTED.

### HEMORRHAGE FROM GUN-SHOT WOUNDS.

In the last number of the *Press* we gave the case of a gun-shot wound of a lad, æt. 14, in whom portions of the entire subclavian artery and vein were shot away, and yet no hemorrhage occurred worth mentioning. Recently, however, we had a case in which death occurred from internal hemorrhage, in consequence of a gun-shot wound, involving some of the branches of the superior mesenteric artery.

Why the difference in the two cases? In the first, the vessel was of immense size and near the heart, while in the latter, the vessels were small and remote from the center of the circulation.

Gun-shot wounds are not, for the most part, followed by much hemorrhage, but an exception to this occurs in wounds involving the abdominal cavity. In gun-shot wounds of the exterior portions of the body, the surrouuding lacerated tissues often embrace, closely, the ends of the torn artery, and thereby prevent hemorrhage, which does not occur in wounds of the mesenteric arteries and their branches. Again, a gun-shot wound involving a vessel belonging to the external parts of the body, will, at once, admit the atmosphere, which, being of a much lower temperature, produces contraction of the ends of the vessels, but which cannot often occur in a gun-shot wound involving the vessels of the abdominal cavity.

In the case above mentioned, involving some of the branches of the superior mesenteric artery, death occurred from hemorrhage, at the end of twenty-four hours after the injury was received. The ball—that of a small pistol—entered the abdominal cavity, midway between the umbilicus and the anterior superior spinous process of the ilium, on the right side, lodged in the left side of the second lumber vertebræ, from the top, taking, in its course, several of the largest branches of the superior mesenteric artery. The blood was evidently discharged from these slowly, as death did not occur for twenty-four hours. The internal hemorrhage was, however, considerably impeded, in consequence of the tightness of a bandage applied around the body, all over the abdomen, which compressed all the abdominal viscera into the smallest compass of which they were capable by this means. This tight bandage was used for the triple purposes of pressing the surrounding parts against the orifices of any communications with the alimentary canal that might have been made by the passage of the ball, and thereby preventing the egress of any of its contents; of preventing hemorrhage, as far as possible, upon the same principles; and, lastly, for the purpose of having the well-known beneficial effects of a tight bandage upon injured parts, to approximate the wounded surfaces and keep them at rest, in order to promote the healing process,

In a post mortem examination of this case, the abdominal cavity was found filled with blood to its utmost capacity, consistent with the tightness of the external pressure. A large coagulam—as large as a man's two fists—was found under the ribs, on the left side, pressing the diaphragm upwards, and considerably out of its natural place. The pelvic cavity was also filled with blood, and clots were found in every recess of the abdominal cavity not influenced by external pressure.—*San Francisco Med. Press.*

